# Differentiated thyroid cancer patients potentially benefitting from postoperative I-131 therapy: a review of the literature of the past decade

**DOI:** 10.1007/s00259-019-04479-1

**Published:** 2019-10-15

**Authors:** Frederik A. Verburg, Glenn Flux, Luca Giovanella, Douglas van Nostrand, Kristoff Muylle, Markus Luster

**Affiliations:** 1 Department of Nuclear Medicine, University Hospital Marburg, Baldingerstraße, 35043 Marburg, Germany; 2 European Association of Nuclear Medicine, Thyroid Committee, Vienna, Austria; 3 Joint Department of Physics, Royal Marsden Hospital and Institute of Cancer Research, Sutton, UK; 4 European Association of Nuclear Medicine, Radiation Protection Committee, Vienna, Austria; 5 Ente Ospedaliero Cantonale, Clinic for Nuclear Medicine and Molecular Imaging and Integrated Centre for Thyroid Diseases, Bellinzona, Switzerland; 6 Washington Hospital Center, Nuclear Medicine, Washington, District of Columbia, USA; 7 Department of Nuclear Medicine, University Hospital Brussels/UZ Brussel (VUB), Brussels, Belgium; 8 European Association of Nuclear Medicine, Board, Vienna, Austria

**Keywords:** I-131 treatment, Prognosis, Recurrence rate, Differentiated thyroid carcinoma

## Abstract

**Background:**

Since the last major review of literature on the benefit of I-131 therapy, the continued debate on postoperative radioiodine treatment (RIT) in differentiated thyroid carcinoma (DTC) has led to a number of further studies being published on this topic.

**Aim:**

The aim of the present paper is to report the results of an updated structured review of the literature pertaining to the prognostic benefits of postoperative RIT in DTC in terms of recurrence-free and disease-specific survival.

**Methods:**

A systematic search of the literature was performed using the Medline and Cochrane Library database. The search period started in August 2007 and ended on December 6, 2017. Search terms used included “differentiated thyroid cancer” and “radioiodine therapy” amended by specific terms for recurrence/disease-free survival or overall and/or cancer-specific survival. Included in the search were systematic reviews, randomized clinical trials, or cohort studies consisting of both patients who underwent postoperative RIT and patients treated by surgery alone.

**Results:**

Eleven retrospective cohort studies met the defined inclusion criteria and were included in the present review. Results of the studies were mixed, with some showing a benefit of RIT even in microcarcinoma whereas others showed no benefit at all.

**Conclusion:**

Literature published in the last decade offers data that support adjuvant postoperative RIT in DTC patients with a tumor diameter exceeding 1 cm. Therefore, at least until randomized prospective studies prove otherwise, the prescription of adjuvant I-131 treatment to all DTC patients with a primary tumor diameter exceeding 1 cm remains a reasonable option.

**Electronic supplementary material:**

The online version of this article (10.1007/s00259-019-04479-1) contains supplementary material, which is available to authorized users.

## Introduction

In recent years, the need for postoperative radioiodine treatment (RIT) in a large number of patients diagnosed with differentiated thyroid carcinoma (DTC) has been questioned ever more fervently. For example, recent guidelines from the American Thyroid Association (ATA) [1] regard this therapeutic modality as something that is not definitely recommended but can merely be “considered” even in classic indications for RIT such as patients with lymph node metastases or limited extrathyroidal extension. Such changes were based on selected evidence produced in a few retrospective series from expert DTC treatment centers, and were not generally accepted [2].

In contrast, a structured review and meta-analysis published by Sawka et al. [3] was able to show a statistically significant benefit in terms of reduction of recurrence rates and the rate of subsequent occurrence of distant metastases in all patients with a tumor diameter > 1 cm. This conforms with studies on the risk of lymph node or distant metastases as a function of tumor diameter which starts to rise from approximately 1 cm [4, 5].

Since the last major review of literature on the benefit of I-131 therapy, the continued debate on postoperative RIT in DTC has led to a number of further studies being published on this topic. In preparation of a quadrilateral meeting of four scientific societies involved in RIT of DTC [6], the aim of the present paper was to provide an updated structured review of the literature pertaining to the prognostic benefits of postoperative RIT in DTC in terms of recurrence-free and disease-specific survival.

## Methods

### Questions

For the purpose of the present review, two explicit questions were defined:

1. Which DTC patients will profit from postoperative RIT in terms of overall and/or cancer-specific survival?
2. Which DTC patients will profit from postoperative RIT in terms of a longer disease-specific survival/low recurrence rates?

### Review of the literature

The systematic search, assessment, and analysis of the literature was performed in December 2017 using the Medline and Cochrane Library database by the “Ärztliches Zentrum für Qualitätsmanagement” (ÄZQ) as commissioned by and in cooperation with the European Association of Nuclear Medicine (EANM). The search period started in August 2007 (end of inclusion of the review by Sawka et al. [3]) and ended on December 6, 2017. Search terms used included “differentiated thyroid cancer” and “radioiodine therapy” amended by specific terms for recurrence/disease-free survival or overall and/or cancer-specific survival. Included in the search were systematic reviews, randomized clinical trials, or cohort studies consisting of both patients who underwent postoperative RIT and patients treated by surgery alone. Furthermore, the reference lists of studies selected for inclusion in the present review were screened for additional relevant reports.

Assessment of the search results was performed in duplicate both by an expert on medical literature assessment from ÄZQ and an expert on thyroid cancer from the EANM (FAV). For further analysis, studies were selected that reported on survival endpoints (overall, cancer-specific, or recurrence-free survival). Based on the observation in the study by Sawka et al. [3] that observation of any possible positive effect of RIT requires a sufficiently large cohort with a sufficiently long follow-up, an empiric selection criterion was defined: studies that included less than 300 patients or reported a follow-up of less than 5 years were excluded.

From the studies identified as relevant, data were extracted on survival, properties of the study population (age, histology, tumor stage), and study design (with a focus on the statistical methodology). A full overview of the search strategy and inclusion and exclusion criteria is given in online supplemental material 1 and 2.

The methodological validity of each study was checked using a structured checklist; grading of the evidence was performed in accordance with the Scottish Intercollegiate Guidelines Network (SIGN) classification, given in the online supplemental material 3.

No further statistical analysis in terms of a meta-analysis was performed as the methodological quality of the studies included was not sufficient to do so correctly with regard to methodology (see also “Results”).

## Results

### Included studies

**Fig. 1:**
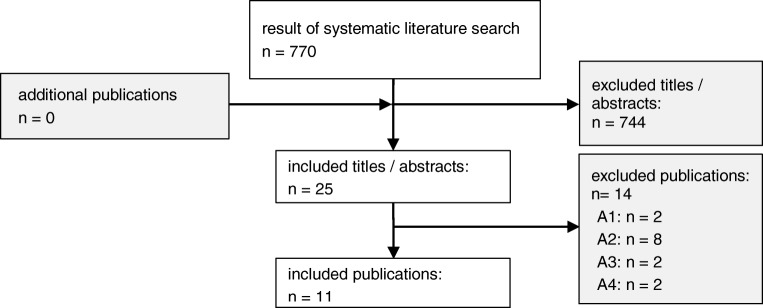
Flowchart of the study selection process. The coded exclusion criteria are explained in the table with exclusion criteria in supplemental material 1

The systematic literature search as described yielded 770 eligible papers. Assessment of title and abstracts led to a review of 26 papers in full-text form. Of these, 11 fulfilled the predefined inclusion criteria. Figure 1 provides a flow-chart of the study selection process. A brief overview of the studies included in this report is given in Table 1. An extensive overview of the extracted relevant data from each study can be found in online supplemental material 4.

**Table 1:** Chronologic overview of the included studies

Ref.	Study	Total *n*	RAI +	Reported outcomes	Data source	Patient cohort
[7]	Kwon 2017	1932	85%	RFS	Seoul registry (1998–2009)	100% microcarcinoma
[8]	Yang 2017	11,832	65–93%	OS (5 years, 10 years)	NCDB database (2002–2012)	100% stage IV
[9]	Zhang 2017	8601	68%	OS; CSS (5 years, 10 years)	SEER database (2004–2013)	100% intermed. risk
[10]	Al-Qahtani 2015	326	56%	DFS (5 years, 10 years)	Riyadh registry (2000–2012)	100% microcarcinoma
[11]	Carhill 2015	4941	74%	OS; DFS	NTCTCSG registry (1987–2012)	43% stage I, 27% II, 24% III (5% IV)
[12]	Kiernan 2015	32,119	24%	OS (5 years, 10 years)	NCDB database (1998–2011)	78% stage I (14% II, 7% III, 1% IV)
[13]	Ruel 2015	21,870	71%	OS	NCDB database (1998–2006)	100% intermed. risk
[14]	Nixon 2013	1129	61%	CSS; RFS (5 years)	MSKCC registry (1986–2005)	41% low risk, 45% intermed. (14% high)
[15]	Kim 2013	704	82%	RFS	Korean registry (1994–2004)	100% microcarcinoma
[16]	Schvartz 2012	1298	70%	OS; DFS (10y)	French registry (1975–2004)	100% low risk
[17]	Lin 2009	7818	22%	OS; CSS	SEER database (1988–2005)	100% microcarcinoma

All studies concern retrospective analyses of single- or multicenter DTC registries. Accordingly, the number of included patients ranges from a few hundred (minimum *n* = 326) to thousands (maximum *n* = 32,119). The fraction of the patient population treated with radioiodine ranged from 22 to 93%.

### Results by stage

#### Microcarcinoma

Of the studies included, four only reported on microcarcinoma (≤ 10 mm without metastases) patients. Two of these four studies showed a statistically significant benefit of I-131 therapy. Al-Qahtani et al. [10] showed a significantly better disease-free survival (DFS) in a Saudi-Arabian population (5-year DFS: RIT 95.7% vs. 92.2% no RIT (unadjusted, *p* = 0.04); 10 year DFS: RIT 90.9% vs. no RIT 84% (unadjusted, *p* = 0.04); multivariate analysis: HR = 0.30 (95%CI 0.2–0.8, *p* < 0.001)). Lin and Bhattacharyya [17] analyzed a group of 7818 patients with papillary microcarcinoma extracted from the Surveillance, Epidemiology and End Results (SEER) database and found a significant positive association between patients receiving RIT (21.5% of the total patient population) and overall survival (RIT 204.3 vs. no RIT 197.5 months; *p* < 0.001). This was retained in multivariate analysis, but the effect was not retained in an analysis of thyroid cancer–specific survival (RIT 214.6 vs. no RIT 212.2 months) so that it is quite possible that further confounders, not analyzed in the study by Lin et al., may have played a role in the positive effect on overall survival.

In contrast, studies by Kim et al. [15] in a population of 740 microcarcinoma patients from Korea and by Kwon et al. [7] in a population of 1932 papillary microcarcinoma cases also from Korea did not show any significant effects of RIT on disease-free, thyroid cancer–specific, or overall survival.

#### Non-microcarcinoma without distant metastases

The patient group with non-microcarcinoma DTC without distant metastases is currently the group of patients in whom adjuvant treatment after thyroidectomy is subject to debate. Six studies examining the benefit of I-131 in this group of patients were identified.

Zhang et al. [18] analyzed 8601 DTC patients with an intermediate risk profile (T1/2 N1 and T3 N0/1) from the SEER database, 67.6% of whom received RIT. Although the authors found a significant favorable influence of RIT on overall survival, also in multivariate analysis, this favorable influence could not be shown for disease-specific survival, again indicating that likely other confounders which may influence the decision on RIT might have played a role but were not reported by the authors.

Carhill et al. [11] analyzed 4941 patients included in the database of the National Thyroid Cancer Treatment Cooperative Study Group (NTCTCS). In this study 74% of patients received postoperative RIT. With regard to overall survival, RIT was identified as a favorable determinant of overall (i.e., analyzing all-cause death) survival in NTCCTS stage III patients only, although not in stages I, II, and IV. In a model combining the extent of surgical resection and RIT, the combination of total thyroidectomy and RIT was also a significant positive determinant of overall survival in stage IV disease. Disease-specific survival was not analyzed. RIT was furthermore a highly significant independent favorable prognostic factor with regard to recurrence-free survival in stage I patients, although not in stage II and III patients.

Ruel et al. [13], also using patients included in the United States National Cancer Database (NCDB), selected 21,870 patients with an intermediate risk profile (≤ 4 cm T1–3 N1 M0/x, >4 cm T3 N0 M0/x). According to the report, 70.5% of patients received adjuvant radioiodine treatment after total thyroidectomy. Multivariate analysis of overall survival showed that RIT was associated with a 29% reduced risk of death (*p* < 0.001). In patients < 45 years of age, RIT was even associated with a 36% reduced risk of death. Again, due to the non-randomized nature of this study, it remains uncertain whether these results were not partially due to a selection bias.

Kiernan et al. [12] performed an interesting study on patients who underwent a one-sided lobectomy as the only surgical procedure and then in the course of disease received RIT as an alternative to completion thyroidectomy. To this end they identified 32,119 patients from the NCDB. Although RIT is not recommended in patients who underwent lobectomy only for tumors with a maximum diameter of 1 cm [1, 19], this procedure was nonetheless performed in 24% of patients included in the study who on average had larger primary tumors and were in a higher average cancer stage. In comparison with those who did not, the patients who underwent RIT in addition to lobectomy had an improved 5- and 10-year survival in spite of a more unfavorable risk profile; multivariate analysis identified RIT as an independent prognostic factor.

In contrast to these four studies documenting favorable effects of RIT on prognosis, a study of 1129 papillary thyroid cancer patients of ATA low-, intermediate-, and high-risk patients from the Memorial Sloan Kettering Cancer Center did not find a positive effect of RIT on disease-specific or recurrence-free survival time [14]. Furthermore, Schvartz et al. [17] in a report on 1298 low-risk patients from two French registries also did not find a beneficial effect of RIT on either overall or disease-free survival.

#### Patients with distant metastases

Although, in patients with iodine avid, advanced DTC there appears to be a general consensus that RIT can be beneficial, it should nonetheless be noted that there is some recent comparative evidence supporting the effectiveness of RIT in these patients. Yang et al. [8] studied 11,832 DTC patients with stage IV DTC from the National Cancer Database. Of these, 67.9% received postoperative RIT. All-cause mortality at 5 and 10 years was nearly doubled in the patients who did not receive RIT compared with those who did undergo this procedure (see table 2). However, some caution is advised as those not receiving RIT were significantly older than those who did, thus providing a source of negative prognostic bias against this group. Nonetheless, in multivariate analyses of each of the TNM stages IVA–IVC, the survival benefit of RIT remained for all histological subgroups (PTC and FTC) in each of these stages.

## Discussion

The review of the more recent literature on the role of RIT in DTC has identified a very similar heterogeneous set of results as the overview and meta-analysis of the literature on this topic by Sawka et al. from 2008. All in all, it seems that the larger the series and the longer the duration of follow-up, the more likely a benefit of RIT is to show.

A major hindrance to the present review is the limited quality of the publications found. The retrospective cohort studies analyzed by definition consist of grade 2 evidence. However, applying the SIGN checklist for cohort studies yields that, based on the heterogeneity of the populations compared, the methodological quality can at best be rated as moderate. These limitations impede a reliable meta-analysis of the data. As is seen in most studies on DTC, the fortunately low DTC-related mortality and recurrence rates hamper statistical analyses as large cohorts and/or long follow-up periods are often required to show the beneficial effects of therapy. Again however, the limited number of studies included have variable follow-up periods and have evaluated different populations with regard to disease stage and prior treatment. It therefore remains difficult in the present review to discern any clear relationship between the follow-up period, study sample size, and outcome.

A quite surprising effect observed in two studies is that the administration of RIT is related to overall survival, but not to DTC-specific survival. Further studies finding a difference in overall survival rates unfortunately do not report on DTC-specific survival, so it remains open whether this is a significant effect or due to statistical uncertainties. Nonetheless, this difference in effect could point to other differences that may include, speculatively, a more intensive follow-up of patients who also received RIT thus allowing for the earlier detection of potentially critical other diseases.

Also, it appears that many confounders may play a role. The quality and extent of surgery may be one of those criteria. It is of course an interesting observation that the prognosis of patients who undergo “radiolobectomy” instead of completion surgery is superior. However, this does not address whether RIT would still improve survival if the patients who required it had, *lege artis*, been treated with surgical total thyroidectomy before undergoing RIT.

On balance, the aggregate of the studies summarized in this overview seems confirmatory of at least some adjuvant effect of RIT in terms of overall survival. On the other hand, it also provides arguments for both sides of the debate pro and contra RIT. None of the studies here provides the answers on whether adjuvant RIT after total thyroidectomy is truly of benefit in terms of patient relevant outcome measures—as any therapy with adjuvant intent should do. The next question is which patients would benefit from RIT. Somewhat surprisingly and contrary to general opinion in the field, in the present study, we even found two studies providing at least some results indicating a positive association between RIT and patient-relevant outcomes in microcarcinoma cases. This requires further study along with whether other factors may be confounding these results including the quality or experience of the surgeons performing the surgery. However, at least in terms of recurrence-free survival even large, methodologically well-documented projects such as the NTCCTS database study [11] find a beneficial effect of postoperative RIT in the lowest risk category—even though this effect was not found for higher stages, possibly due to much lower patient numbers, especially of those not receiving RIT, in the latter groups.

## Conclusion

Literature published in the last decade offers data that support adjuvant postoperative RIT in DTC patients with a tumor diameter exceeding 1 cm. Therefore, at least until randomized prospective studies prove otherwise, the prescription of adjuvant I-131 treatment to all DTC patients with a primary tumor diameter exceeding 1 cm remains a reasonable option.

## Electronic supplementary material

ESM 1 (DOC 215 kb)
