# Supplementary material for: Differentiated thyroid cancer patients potentially benefitting from postoperative I-131 therapy: a review of the literature of the past decade
Source: Eur J Nucl Med Mol Imaging. 2019 Oct 15;47(1):78–83. doi: 10.1007/s00259-019-04479-1 (PMC6885024; doi:10.1007/s00259-019-04479-1)
Supplement: Supplementary file 1 — (DOC 215 kb) [file 259_2019_4479_MOESM1_ESM.doc]

## Supplemental material

**Differentiated thyroid cancer patients potentially benefitting from postoperative I-131 treatment: a review of the literature of the past decade**

Frederik A. Verburg (1,6), Glenn Flux (2,7), Luca Giovanella (3,6), Douglas van Nostrand (4,9), Kristoff Muylle (5,8), Markus Luster (1,6)

1: University Hospital Marburg, Department of Nuclear Medicine, Marburg, Germany

2: Joint Department of Physics, Royal Marsden Hospital and Institute of Cancer Research, Sutton, UK.

3: Ente Ospedaliero Cantonale, Clinic for Nuclear Medicine and Molecular Imaging and Integrated Centre for Thyroid Diseases, Bellinzona, Switzerland

4: Washington Hospital Center, Nuclear Medicine, Washington, District of Columbia, United States

5: Department of Nuclear Medicine, University Hospital Brussels / UZ Brussel (VUB), Brussels, Belgium.

6: European Association of Nuclear Medicine, Thyroid Committee

7: European Association of Nuclear Medicine, Radiation Protection Committee

8: European Association of Nuclear Medicine, Board

9: Society of Nuclear Medicine and Molecular Imaging

## 1. In- and exclusion criteria

| **Population** | **patients with differentiated thyroid carcinoma** (DTC; papillary or follicular; also: polydifferentiated or insulary) |
| --- | --- |
| Intervention | I-131 as postoperative, adjuvant therapy |
| Control | no I-131 (operation only) |
| end points | overall and cancer-specific survival; recurrence-free survival (follow-up at least 5y) |
| study types | comparing cohort studies (prospective registries) |
| Languages | english or german |

1. **exclusion criteria (abstract assessment, full text assessment)**

| **A1** | **other disease / other type of cancer; other intervention** |
| --- | --- |
| A2 | No control group or not analyzed / no report of pre-defined end points; median follow-up <5y (60mo) |
| A3 | Other publication or study type (narrative review, editorial; case report, case series) // methodologically insufficient (i.e. no adjusted analyses) |
| A4 | study population too small (n<300; or one of the RAIT groups n<100) |

## 2. Search strategies

Search strategy in Pubmed on december 6, 2017

| no. | Search question | results |
| --- | --- | --- |
| #8 | #7 Filters: Publication date from 2007/08/01 | **796** |
| #7 | #6 AND ((((((((("Case-Control Studies"[Mesh] OR "Cohort Studies"[Mesh]))) OR "Cross-Sectional Studies"[Mesh]) OR (("Follow-Up Studies"[Mesh] OR (("follow-up" OR "follow up") AND (studies OR study))))) OR ((longitudinal[tw] OR retrospective[tw] OR "cross-sectional"[tw] OR "cross sectional"[tw]))) OR ("case control"[tw] OR (cohort[tw] AND analy*[tw]))) OR "Observational Study"[pt]))) | 1446 |
| #6 | #5 AND (surviv*[tiab] OR mortalit*[tiab] OR death[tiab] OR relaps*[tiab] OR recurr*[tiab] OR progress*[tiab]) | 3034 |
| #5 | #3 AND #4 | 7882 |
| #4 | RAI[tiab] OR RAIT[tiab] OR RRA[tiab] OR "radioactive iodine"[tiab] OR "I-131"[tiab] OR "I131"[tiab] OR “131I”[tiab] OR “131-I”[tiab] OR radioiodine[tiab] | 29004 |
| #3 | #1 OR #2 | 68002 |
| #2 | (thyroid[tiab] AND (cancer*[tiab] OR carcinom*[tiab] OR neoplas*[tiab] OR tumor*[tiab] OR tumour*[tiab])) | 56646 |
| #1 | "Thyroid Neoplasms"[Mesh] | 46734 |

**Search strategy in the Cochrane Library on december 6,** 2017

| no. | Search question | results |
| --- | --- | --- |
| #8 | #7 in Cochrane Reviews (Reviews only), Other Reviews and Trials  Publication Year from 2007 to 2017 | **77** |
| #7 | #6 not “conference abstract”:pt | 104 |
| #6 | #5 and (surviv*:ti,ab,kw or mortalit*:ti,ab,kw or death:ti,ab,kw or relaps*:ti,ab,kw or recurr*:ti,ab,kw or progress*:ti,ab,kw) | 169 |
| #5 | #3 and #4 | 364 |
| #4 | RAI:ti,ab,kw or RAIT:ti,ab,kw or RRA:ti,ab,kw or "radioactive iodine":ti,ab,kw or "I-131":ti,ab,kw or "I131":ti,ab,kw or "131I":ti,ab,kw or "131-I":ti,ab,kw or radioiodine:ti,ab,kw | 1045 |
| #3 | #1 or #2 | 1570 |
| #2 | thyroid:ti,ab,kw and (cancer*:ti,ab,kw or carcinom*:ti,ab,kw or neoplas*:ti,ab,kw or tumor**:ti,ab,kw or tumour*:ti,ab,kw) | 1484 |

- Cochrane Database of Systematic Reviews (6)
- Other Reviews (4)
- Cochrane Central Register of Controlled Trials (67)
- Cochrane Methodology Register (not searched)
- Health Technology Assessment Database (not searched)
- NHS Economic Evaluation Database (not searched)

## 3. Grading of evidence

The included studies were assessed for methodological quality using the Scottish Intercollegiate Guidelines Network (SIGN) classification (<http://www.sign.ac.uk/guidelines/fulltext/50/annexoldb.html>).

| grade | description |
| --- | --- |
| 1++ | High-quality meta-analyses, high-quality systematic reviews of clinical trials with very little risk of bias |
| 1+ | Well-conducted meta-analyses, systematic review of clinical trials or well-conducted clinical trials with low risk of bias |
| 1- | Meta-analyses, systematic reviews of clinical trials or clinical trials with high risk of bias |
| 2++ | High-quality systematic reviews of cohort or case and control studies; cohort or case and control studies with very low risk of bias and high probability of establishing a causal relationship |
| 2+ | Well-conducted cohort or case and control studies with low risk of bias and moderate probability of establishing a causal relationship |
| 2- | Cohort or case and control studies with high risk of bias and significant risk that the relationship is not causal |
| 3 | Non-analytical studies, such as case reports and case series |
| 4 | Expert opinion |

The intermediate classification (+/-) which was used in the grading of several papers included in the evidence table indicates that the risk of bias and the likelihood of a causal relationship was estimated as **moderate**.

## 4. Table of evidence of included studies

Table 1: Table of evidence

| **reference** | **population** | **intervention** | **study design** | **results** | **methodological validity (adapted from SIGN)** | **level of evidence** |
| --- | --- | --- | --- | --- | --- | --- |
| **studies on differentiated thyroid carcinoma (all stages / risk groups)** | | | | | | |
| Yang Z.  Comparison of Survival Outcomes Following Postsurgical Radioactive Iodine Versus External Beam Radiation in Stage IV Differentiated Thyroid Carcinoma.  Thyroid 2017  [1] | differentiated thyroid carcinoma, n=11,832  PTC: 91.24%  FTC: 8.76%  pat.s with dx of stage IV; thyroidectomy as primary surg.tx  mean age 61.6y | adjuvant radiation tx: RAI, EBRT, or no RT  - PTC: RAI+/- n=7500 vs 2692  - FTC: RAI+/- n=538 vs 286  median follow-up [unclear - data for 5y and 10y] | National Cancer Database (tx in 2002-2012)  stratification by histology (follicular vs papillary) and sub-stage  [multivariable models - adjusted for age, sex, race, socioecon. factors; clinical variables: surgical length of stay, tx w/ neck dissection -- *adjusted analyses for sub-stages only*]  tumor staging system: according to AJCC | cohort group characteristics:  PTC cohort - significant differences: group +RAI younger than -RAI group (median 58 vs 61y)  FTC cohort - significant differences: group +RAI younger than -RAI group (median 66 vs 73y)  all-cause mortality:  PTC cohort  - 5y mortality: 22.7% w/o RAI vs 11.0% with adjuvant RAI  - 10y mortality: 25.5% w/0 RAI vs 14.0% with adjuvant RAI  FTC cohort  - 5y mortality: 45.5% w/o RAI vs 29.2% with adjuvant RAI  - 10y mortality: 51% w/o RAI vs 36.8% with adjuvant RAI  hazards of death calculated for sub-stages (IV-A, -B, -C) but "*more than 70% of pat.s had missing grade data. the authors therefore advise caution when interpreting the significance of tumor grade data in this study."* | study type? - retrospective NCDB analysis  study population described (table)? - yes  population well-balanced? - no ("age was found to be significantly related to higher death hazards" -- mortality rates not adjusted for this factor)  drop-out reported (consort diagram)? - no / n.a.  statistic methods reported? - yes  definition of endpoint parameters/ treatment protocol reported? - yes (stages / classification)  ITT analysis? - n.a.  financial support/conflicts of interest reported? which? - yes (none) | 2-  (unclear whether rates adjusted for full cohort; multivar. analyses for substages but many missing data, therefore high risk of bias) |
| Zhang H.  Postoperative radioactive iodine-131 ablation is not necessary among patients with intermediate-risk differentiated thyroid carcinoma: a population-based study. Hell J Nucl Med 2017  [2] | differentiated thyroid carcinoma, n=8,601  PTC: 83.6%  FTC: 16.4%  pat.s with intermediate risk (T1/2 N1 M0 and T3 with/ without N1 M0)  T1/T2: 27.3%  T3: 72.7%  mean age 47.3y | adjuvant RAI (in 67.6% of cohort pat.s)  - 68.8% of PTC  - 61.6% of FTC  - T1/T2:  25% no RAI, 28.3% +RAI  median follow-up 10.3y | SEER database (2004-2013)  univariate analyses to identify risk factors -> Cox multivariate regression analysis  tumor staging system: according to AJCC TNM classification system | cohort group characteristics:  significant differences: +RAI group more white pat.s, more PTC, more with tumor size of T1/T2 compared with -RAI group; fewer T3 tumors, fewer N0, fewer localized tumors without ETE, fewer solitary tumors (all p<0.001). (mean age 47 vs 48y, p=0.008)  *<-- multivariate analysis: all factors associated with either OS and/or CSS but no matching (only stratification for T category)*  overall survival:  5y-OS rates: 95.0% vs 96.8% (- vs +) // 10y-OS rates: 89.8% vs 92.2%  mean OS in -RAI group 112.9mo vs 114.9mo in +RAI group (p<0.001; absolute difference 2mo)  subgroup analyses (univariate) for tumor size:  - T1/T2: not significant (-RAI 114.4mo vs 115.8mo +RAI; p=0.113)  - T3: significant diff. (-RAI 112.4mo vs 114.7mo +RAI; p<0.001)  multivariate analysis (full cohort, T1-T3):  significant benefit of RAI, HR=0.71 (95%CI 0.56-0.90; p=0.004)  no effect of T3 in multivar.analysis --> other factors likely causal  thyroid cancer-specific death:  5y-CSS rates: 98.8% vs 99.2% (- vs +) // 10y-OS rates: 98.2% vs 97.8%  mean CSD in -RAI group 117.7mo vs 118.0mo in +RAI (p=0.164)  subgroup analyses (univariate) for tumor size:  - T1/T2: not significant (-RAI 118.6mo vs 118.5mo +RAI; p=0.801)  - T3: not significant (-RAI 117.3mo vs 117.8mo +RAI; p=0.154)  *[no multivar.analysis for RAI effect on CSS because no diff. in univar.]* | study type? - retrospective SEER analysis  study population described (table)? - yes  population well-balanced? - no (but stratified and multivariate analyses)  drop-out reported (consort diagram)? - yes (according to criteria) / n.a.  statistic methods reported? - yes  definition of endpoint parameters/ treatment protocol reported? - yes (classification)  ITT analysis? - n.a.  financial support/conflicts of interest reported? which? - yes (none) | 2(+/-)  (stratified and multivar. analyes for hazard ratios) |
| Carhill AA.  Long-Term Outcomes Following Therapy in Differentiated Thyroid Carcinoma: NTCTCS Registry Analysis 1987-2012.  J Clin Endocrinol Metab 2015  [3] | differentiated thyroid carcinoma, n=4,941  PTC 88%  FTC 8%  HTC 4%  stage I: 43%,  II: 27%, III: 24%,  IV: 5%  mean age: not reported (52% <45y, 48% >45y) | adjuvant RAI (in 74% of cohort pat.s)  median follow-up 6y | National Thyroid Cancer Treatment Cooperative Study Group registry analysis (11 institutions)  (dx between 1987-2012)  tumor staging system: according to registry staging system (I-IV) | cohort group characteristics:  significant differences between +-RAI unclear /not shown  overall survival:  multivariate analyses, per stage:  - stage I: +RAI vs -RAI risk ratio RR=0.79 (95%CI 0.35-1.89; p=0.58)  - stage II: +RAI vs -RAI RR=0.67 (95%CI 0.36-1.28; p=0.22)  - stage III: +RAI vs -RAI RR=0.66 (95%CI 0.46-0.98; p=0.04) signif.  - stage IV: +RAI vs -RAI RR=0.70 (95%CI 0.46-1.10; p=0.12)  disease-free survival:  - stage I: +RAI vs -RAI risk ratio RR=1.79 (95%CI 1.28-2.56; p<0.001)  - stage II: +RAI vs -RAI RR=0.70 (95%CI 0.49-1.01; p=0.53)  - stage III: +RAI vs -RAI RR=0.84 (95%CI 0.57-1.28; p=0.40)  “Initial disease stage was a significant predictor of OS as well as DFS. No significant difference was observed in analysis of treatment outcomes among the histological subtypes.” | study type? - retrospective registry analysis  study population described (table)? - no (not for +/- RAI groups)  population well-balanced? - no / unclear  drop-out reported (consort diagram)? - yes  statistic methods reported? - yes  definition of endpoint parameters/ treatment protocol reported? - yes (classification: see supplement)  ITT analysis? - no / n.a.  financial support/conflicts of interest reported? which? - yes (NTCTCS has been supported in part by research grants from Genzyme / Sanofi company, and Pfizer, and  by the University of Texas) | 2-  (many data missing for assessment) |
| Kiernan CM.  Use of radioiodine after thyroid lobectomy in patients with differentiated thyroid cancer: does it change outcomes?  J Am Coll Surg 2015  [4] | differentiated thyroid carcinoma, n=32,119  pat.s with thyroid lobectomy as definitive procedure  PTC: 83%  FTC: 11%  Hürthle: 6%    stage I: 78%,  II: 14%, III: 7%,  IV: 1%  mean age 48y | adjuvant RAI (in 24% of cohort pat.s)  median follow-up 81-86mo | National Cancer Database (dx between 1998-2011)  Cox proportional hazards regression modeling to identify factors independently associated with OS; multivariable regression analysis  tumor staging system:  coded according to the NCDB analytic stage group (value of reported pathologic stage group / clinical stage group if pathol. stage is not available) | cohort group characteristics:  significant differences: pat.s +RAI younger (47 vs 49y; more FTC (18% vs 9%) and HCC (8% vs 5%) and fewer PTC (74% vs 86%) in the RAI cohort; +RAI pat.s presented at later stages (stage I 82% vs 62% +RAI, stage II 12% vs 23% +RAI, stage III 5% vs 12% +RAI), had larger cancers (2.2 vs 0.7cm), more likely with ETE (9% vs 3%) and positive LN (25% vs. 10%).  overall survival:  - unadjusted analysis: OS slightly greater in +RAI group at 5y (97% vs. 95%, p<0.001) and 10y (91% vs. 89%, p<0.001)  - regression analysis (adjusting for multiple patient, tumor and hospital factors): statistically significant survival benefit in +RAI group (HR=0.53, 95%CI 0.38–0.72; p<0.001)  [no subgroup analyses for stages]  factors significantly associated with decreased OS: age ≥45, male gender, black race, Medicaid insurance, Medicare insurance, tumor size ≥ 1cm, extrathyroidal extension, distant metastases | study type? - retrospective NCDB analysis  study population described (table)? - yes  population well-balanced? - no (but multivariate analysis)  drop-out reported (consort diagram)? - no / n.a.  statistic methods reported? - yes  definition of endpoint parameters/ treatment protocol reported? - (brief) (stages / classification)  ITT analysis? - n.a.  financial support/conflicts of interest reported? which? - yes (none) | 2(+-)  (adjusted multivar. analyses for hazard ratio) |
| Ruel E.  Adjuvant radioactive iodine therapy is associated with improved survival for patients with intermediate-risk papillary thyroid cancer.  J Clin Endocrinol Metab 2015  [5] | differentiated (papillary) thyroid carcinoma, n=21,870  adult pat.s with thyroidectomy; intermediate risk (≤4cm, T1-3 N1 M0/x or >4cm T3 N0 M0/x)  mean age 43/44y | adjuvant RAI (in 70.5% of cohort pat.s)  mean follow-up 6.8y / median 6.6y | National Cancer Database (dx between 1998-2006)  multivariate Cox proportional hazards model to examine OS after adjustment for clinical and demographic factors across all ages and in atients aged younger than 45 years  tumor staging system: according to ATA risk criteria & AJCC staging | cohort group characteristics:  significant differences: +RAI group more multifocal tumors (51% vs 47%), lymph node involvement (74% vs 68%), and positive surgical margin status (19% vs 15%)  overall survival:  (most patients were alive as of 2006, and a median survival time could not be estimated.)  multivariate analysis (adjustment for demogr. and clin. factors): RAI associated with a 29% reduced risk of death (HR=0.71; 95%CI 0.62-0.82, p<0.001)  - patient factors associated with compromised OS: older age,  male gender, black race  - pathol. and clin. factors: larger tumor size, presence of lymph node metastases, positive surgical margins, lack of RAI therapy  subgroup analysis für pat.s <45y: RAI associated with a 36% reduced risk of death (HR=0.64; 95%CI 0.45-0.92; p=0.016)  - patient factors associated with compromised OS: male gender | study type? - retrospective NCDB analysis  study population described (table)? - yes  population well-balanced? - no (but multivariate analysis)  drop-out reported (consort diagram)? - yes (according to criteria)  statistic methods reported? - yes  definition of endpoint parameters/ treatment protocol reported? - yes (classification)  ITT analysis? - n.a.  financial support/conflicts of interest reported? which? - yes (none) | 2(+-)  (adjusted multivar. analyses for hazard ratio) |
| Nixon IJ.  The results of selective use of radioactive iodine on survival and on recurrence in the management of papillary thyroid cancer, based on Memorial Sloan-Kettering Cancer Center risk group stratification.  Thyroid 2013  [6] | differentiated, papillary thyroid carcinoma, n=1,129  pT1: 45%  pT2: 16%  pT3: 32%  pT4: 7%  ATA risk groups: 41% low, 45% intermediate, 14% high  median age 46y | adjuvant RAI (in 61% of cohort pat.s)  - low-risk 21% (vs 72% -RAI)  - interm. 60% (vs 22% -RAI)  - high 19% (vs 6% -RAI)  [% of entire cohort]  median follow-up 63mo | Memorial Sloan-Kettering Cancer Center registry analysis (1986-2005)  multivariate analyses performed [with limited variables]  tumor staging system: according to MSKCC cause-specific mortality risk classification system, and ATA stratification system | cohort group characteristics:  significant differences: +RAI group more likely male, more advanced pT and N stage disease, more likely in high-risk group  disease-specific survival:  univariate analysis: no predictive effect of RAI on DSS (5y rates +RAI 99% vs 100% -RAI, p=0.821)  (multivariate analysis for DSS only possible for age, pT stage; not significant for +/-RAI [no HR reported])  recurrence-free survival:  univariate analysis: worse 5y-RFS with RAI than w/o (90% vs 97%) but not in multivariate analysis (incl. age, sex, T, N, RAI)  subgroup analyses:  T1/T2 N0/Nx: n=490 (178 vs 312); few events (5y-DSS 100% vs 98%, 5y-RFS 99% vs 100%), not significantly different  T1/T2 N1: n=193 (142 vs 51), few events (5y-regional-RFS 93% vs 100%, 5y-distant-RFS 93% vs 100%), not significanty different  T3/T4: n=444 (371 vs 73), significant difference in 5y-regional-RFS (+RAI 91% vs 98% -RAI, p=0.019), 5y-distant-RFS not significantly different (both 93%) | study type? - retrospective registry analysis  study population described (table)? - yes  population well-balanced? - no (multivar. analysis with few variables)  drop-out reported (consort diagram)? - yes (according to criteria)  statistic methods reported? - yes  definition of endpoint parameters/ treatment protocol reported? - yes (classification)  ITT analysis? - n.a.  financial support/conflicts of interest reported? which? - yes (none) | 2(+-)  (multivar. Analyses, with few variables) |
| Schvartz C.  Impact on overall survival of radioactive iodine in low-risk differentiated thyroid cancer patients.  J Clin Endocrinol Metab 2012  [7] | differentiated thyroid carcinoma n=1,298  PTC: 72%  FTC: 28%  pat.s with low risk  - pT1: 62%  - pT2: 38%  mean age at dx 46.6y ±14y | adjuvant RAI (in 70% of cohort pat.s)  median follow-up 10.3y | retrospective cohort study (2 French registries)  (tx between 1975-2004)  univariate and multivariate Cox analyses, analyses stratified on propensity score (age, sex, extent of surgery, node surgery, histology, pT)  tumor staging system: according to ATA and ETA criteria | cohort group characteristics:  significant differences: +RAI group older, mostly thyroidectomy, less pN0 (47% vs 73%), more papillary (77% vs 62%), less pT1 (59% vs 68%)  overall survival:  unadjusted analysis: 10y OS +RAI 94.6% vs 95.8% -RAI (p<0.01)  adjusted on propensity score: not significantly different (p=0.35); adjusted univariate HR=0.75 (95%CI 0.40-1.38) for RAI  disease-free survival:  unadjusted analysis: 10y DFS +RAI 88.7% vs 93.1% -RAI (p<0.01)  adjusted on propensity score: not significantly different (p=0.48). adjusted univariate HR=1.11 (95%CI 0.73-1.70) for RAI  “based on multivariate Cox analysis, age and sex were the only two independent prognostic factors associated with DFS and OS. If RAI had a significant and deleterious effect on survival in univariate analysis, this effect disappeared after adjustment was performed on the covariates in the multivariate analysis.” | study type? - retrospective registry analysis  study population described (table)? - yes  population well-balanced? - no (but propensity score adjustments)  drop-out reported (consort diagram)? - n.a.  statistic methods reported? - yes  definition of endpoint parameters/ treatment protocol reported? - yes (classification)  ITT analysis? - n.a.  financial support/conflicts of interest reported? which? - yes (none) | 2(+-)  (adjusted multivar. analyses for hazard ratio) |
| **studies on thyroid microcarcinoma** | | | | | | |
| Kwon H.  Lack of Efficacy of Radioiodine Remnant Ablation for Papillary Thyroid Microcarcinoma: Verification Using Inverse Probability of Treatment Weighting.  Ann Surg Oncol 2017  [8] | papillary microcarcinoma, n=1,932  pat.s without lateral cervical LN or distant metastasis, undergoing total thyroidectomy  [stages: unclear]  mean age 50y | adjuvant RAI (in 85.3% of cohort pat.s)  median follow-up 8.3y | retrospective cohort study (Seoul, 1998-2009)  weighted regression analysis adjusting for risk factors (age, sex, tumor size, ETE, multifocality, central cervical metastas.)  [TNM system: unclear / n.a.] | cohort group characteristics:  significant differences: +RAI group larger primary tumor size (0.7 vs 0.5cm), higher percentage of ETE (55% vs 33%), cervical lymph node metastasis (34% vs 4%)  recurrence-free survival:  univariate analysis: no significant difference [% not reported]  adjusted analysis (different models): no association of RAI with RFS (HR 0.90 / 1.03 / 2.02; wide confidence intervals)  regression analysis - predictive factors:  older age and female gender associated with better RFS;  larger primary tumor size, ETE, multifocal tumor, cervical LN metastasis significantly associated with increased recurrence | study type? - retrospective cohort analysis  study population described (table)? - yes  population well-balanced? - no (but adjusted analyses)  drop-out reported (consort diagram)? - yes / n.a.  statistic methods reported? - yes  definition of endpoint parameters/ treatment protocol reported? - yes  ITT analysis? - n.a.  financial support/conflicts of interest reported? which? - yes (none) | 2(+-)  (adjusted multivar. analyses for hazard ratio) |
| Al-Qahtani KH.  Adjuvant Radioactive iodine 131 ablation in papillary microcarcinoma of thyroid: Saudi Arabian experience [corrected].  J Otolaryngol Head Neck Surg 2015  [9] | papillary microcarcinoma, n=326  - classic: 81.3%  - follicular: 12.6%  - tall cell: 3.4%  - Hürthle: 2.5%  stage I: 66.5%  stage III: 29.5%  stage IV: 4%  mean age at dx 42.6y (±11.6) | adjuvant RAI (in 55.8% of cohort pat.s)  median follow-up 8y | retrospective, bicentric study (Riyadh, 2000-2012)  multivariate regression analysis to identify prognostic factors  tumor staging system: according to AJCC | cohort group characteristics:  significant differences: +RAI group larger tumor size (0.72 vs 0.44cm); less classic variants; more likely multifocal; higher percentage of ETE, LVSI, surgical margins, LN metastasis, stages III&IV  disease-free survival:  5y-DFS: +RAI 95.7% vs 92.2% -RAI (unadjusted, p=0.04)  10y-DFS: +RAI 90.9% vs 84% -RAI (unadjusted, p=0.04)  multivariate analysis: HR=0.30 (95%CI 0.2-0.8, p<0.001)  regression analysis - prognostic factors:  histopathologic variants, multifocality, ETE, nodal status, and adjuvant RAI ablation (all p<0.001) | study type? - retrospective cohort analysis  study population described (table)? - yes  population well-balanced? - no (but multivar. analyses for predictive factors)  drop-out reported (consort diagram)? - yes / n.a.  statistic methods reported? - yes  definition of endpoint parameters/ treatment protocol reported? - yes(stages / classification)  ITT analysis? - n.a.  financial support/conflicts of interest reported? which? - yes (none) | 2(+-)  (adjusted multivar. analyses for hazard ratio) |
| Kim HJ.  Radioactive iodine ablation does not prevent recurrences in patients with papillary thyroid microcarcinoma.  Clin Endocrinol (Oxf) 2013  [10] | papillary microcarcinoma,  n=704  pat.s with total thyroidectomy (considered disease-free)  - low-risk: 32%  - interm.risk: 68%  (stage I: 63%,  III: 23%, IV: 14%)  mean age at dx 47y (±11) | adjuvant RAI (in 82% of cohort pat.s)  median follow-up 64mo | retrospective cohort study (Korea, 1994-2004)  propensity score included gender, age, tumour size, ETE, cervical LN metastasis and tumor multifocality  (TNM) staging according to UICC / AJCC (7th edition) | cohort group characteristics:  significant differences: +RAI group larger primary tumor size, more likely multifocal (37% vs 12%), ETE (53% vs 15%), cervical LN metastasis (28% vs 4%); less stage I (57% vs 89%)  - *subgroup characteristics reported for intermediate-risk (n=480): hardly significant differences, but very small control group (n=30)*  recurrence-free survival:  - unadjusted analysis, intermediate-risk: not significantly different (p=0.52; n=6 recurrences in +RAI group)  - multivariate analysis, all pat.s: not significantly different (p=0.17); intermediate-risk: not significantly different (p=0.79)  *[no hazard ratios calculated / reported]* | study type? - retrospective cohort analysis  study population described (table)? - yes  population well-balanced? - no (but multivar. analysis)  drop-out reported (consort diagram)? - yes  statistic methods reported? - yes  definition of endpoint parameters/ treatment protocol reported? - yes (stages / classification)  ITT analysis? - n.a.  financial support/conflicts of interest reported? which? - yes (none) | 2(+-)  (adjusted multivar. analyses) |
| Lin HW.  Survival impact of treatment options for papillary microcarcinoma of the thyroid.  Laryngoscope 2009  [11] | papillary microcarcinoma, n=7,818  [stages: unclear]  mean age at dx 48.6y | adjuvant RAI (in 21.5% of cohort pat.s)  [mean follow-up unclear; 5y and 10y survival rates reported] | SEER database (1988-2005)  multivariable regression models (variables analyzed: extent of thyroidectomy, use of RAI, age, sex)  [TNM system: unclear / n.a.] | cohort group characteristics:  not shown  overall survival:  univariate analysis: significant benefit of RAI (204.3 vs 197.5mo, p<0.001)  multivariate analysis: negative effect of age at dx and male sex, positive effect of RAI tx [no hazard ratios shown]  disease-specific survival:  univariate analysis: no signif. benefit of RAI (214.6 vs 212.2mo)  multivariate analysis: negative effect of age at dx | study type? - retrospective SEER analysis  study population described (table)? - no  population well-balanced? - unclear  drop-out reported (consort diagram)? - no / n.a.  statistic methods reported? - yes (brief)  definition of endpoint parameters/ treatment protocol reported? - no  ITT analysis? - n.a.  financial support/conflicts of interest reported? which? - yes (none) | 2-  (many data missing for assessment) |

Table 2: Overview of the systemic review by Sawka et al., 2008

| **reference** | **population** | **search strategy, inclusion criteria** | **included studies** | **results** | **methodological validity (AMSTAR)** | **level of evidence** |
| --- | --- | --- | --- | --- | --- | --- |
| Sawka AM.  An Updated Systematic Review and Commentary Examining the Effectiveness of Radioactive Iodine Remnant Ablation in Well-Differentiated Thyroid Cancer.  Endocrinol Metab Clin N Am 2008  [12] | studies (RCTs or cohort studies) on adult patients with  - well differentiated thyroid cancer (papillary, follicular  or follicular variant of papillary)  - surgical treatment involving bilateral resection, (total, near-total, or subtotal thyroidectomy  - radioactive iodine ablation within 1yr after the operation  - median or mean follow-up period of at least 5 years  - reporting outcomes of any cancer-related deaths, cancer recurrence, local-regional recurrence in the thyroid bed or regional lymph nodes, or distant metastases (all at 10 years for data unadjusted for prognostic factors or interventions) | updated search for the time period spanning from the prior review (original search in late 2002) until August 2007.  databases searched:  Medline and other nonindexed citations, the Cochrane Database for Systematic Reviews, Database of Abstracts and Reviews, the Controlled Clinical Trials Database, American College of Physicians Journal Club, the Cochrane Clinical Trials Registry, Embase | all studies in the original systematic review: n=21, plus n=7 additional, new studies  (no long-term randomized, controlled trials examining thyroid cancer-related outcomes after RRA identified --> restricted to observational data) | systematic review of studies with adjustment for prognostic factors (no meta-analysis due to heterogenity):  - 3 out of 12 adjusted studies observed a significant benefit of RRA on the risk of thyroid cancer-related mortality  - 3 out of 6 adjusted studies observed a significant benefit of RAI on the risk of any thyroid cancer recurrence  - significant benefit of RAI on distant metastatic recurrence in 2 adjusted studies with papillary pat.s but not in 1 study with exclusively follicular pat.s  meta-analysis of pooled studies without adjustments:  (- no data on mortality / survival reported)  - statistically significant heterogeneity of RRA on the outcome of any recurrence, precluding meaningful estimation of an overall treatment effect  “Upon carefully examining the best existing long-term observational evidence, the authors could not confirm a significant, consistent, benefit of RRA in decreasing cause specific mortality or recurrence in early stage WDTC. RRA use was associated with a significantly decreased risk of distant metastases; however, this event was relatively rare in papillary cancer. The relatively low risk of thyroid cancer-related death in early stage thyroid carcinoma patients may limit the ability to prove a significant treatment benefit for this outcome.” | 1. a priori Design? - yes (update of prior syst. review)  2. selection and extraction by two independent reviewers? - yes  3. literature search min 2 databases + 1 add.source? - yes (5 db + DARE + Cochrane Clin.Trials db)  4. grey literature incl. - no  5. full list of incl. and excl. studies? - no  6. details of incl. studies? - yes  7. quality of incl. studies assessed? - no  8. reference to method. quality in conclusion? - no  9. heterogenity assessed?- yes  10. publication bias assessed (funnel plot)? - no  11. CoI / funding for review and all studies reported? - no  AMSTAR score 5/11 | 2 |

1. Yang Z, Flores J, Katz S, Nathan C-A, Mehta V. Comparison of Survival Outcomes Following Postsurgical Radioactive Iodine Versus External Beam Radiation in Stage IV Differentiated Thyroid Carcinoma. Thyroid 2017;27:944–52.

2. Zhang H, Cai Y, Zheng L, Zhang Z, Jiang N. Postoperative radioactive iodine-131 ablation is not necessary among patients with intermediate-risk differentiated thyroid carcinoma: a population-based study. Hell J Nucl Med 2017;20:3–10.

3. Carhill AA, Litofsky DR, Ross DS, Jonklaas J, Cooper DS, Brierley JD, et al. Long-Term Outcomes Following Therapy in Differentiated Thyroid Carcinoma: NTCTCS Registry Analysis 1987–2012. J Clin Endocrinol Metab 2015;100:3270–9.

4. Kiernan CM, Parikh AA, Parks LL, Solórzano CC. Use of radioiodine after thyroid lobectomy in patients with differentiated thyroid cancer: does it change outcomes? J Am Coll Surg 2015;220:617–25.

5. Ruel E, Thomas S, Dinan M, Perkins JM, Roman SA, Sosa JA. Adjuvant Radioactive Iodine Therapy Is Associated With Improved Survival for Patients With Intermediate-Risk Papillary Thyroid Cancer. J Clin Endocrinol Metab. 2015;100:1529–36.

6. Nixon IJ, Ganly I, Patel SG, Palmer FL, Di Lorenzo MM, Grewal RK, et al. The Results of Selective Use of Radioactive Iodine on Survival and on Recurrence in the Management of Papillary Thyroid Cancer, Based on Memorial Sloan-Kettering Cancer Center Risk Group Stratification. Thyroid 2013;23:683–94.

7. Schvartz C, Bonnetain F, Dabakuyo S, Gauthier M, Cueff A, Fieffé S, et al. Impact on overall survival of radioactive iodine in low-risk differentiated thyroid cancer patients. J Clin Endocrinol Metab 2012;97:1526–35.

8. Kwon H, Jeon MJ, Kim WG, Park S, Kim M, Kim TY, et al. Lack of Efficacy of Radioiodine Remnant Ablation for Papillary Thyroid Microcarcinoma: Verification Using Inverse Probability of Treatment Weighting. Ann Surg Oncol 2017;24:2596–602.

9. AL-Qahtani KH, Al Asiri M, Tunio MA, Aljohani NJ, Bayoumi Y, Fatani H, et al. Adjuvant Radioactive iodine 131 ablation in papillary microcarcinoma of thyroid: Saudi Arabian experience. J Otolaryngol - Head Neck Surg 2015;44:51.

10. Kim HJ, Kim NK, Choi JH, Kim SW, Jin S-M, Suh S, et al. Radioactive iodine ablation does not prevent recurrences in patients with papillary thyroid microcarcinoma. Clin Endocrinol (Oxf) 2013;78:614–20.

11. Lin HW, Bhattacharyya N. Survival impact of treatment options for papillary microcarcinoma of the thyroid. Laryngoscope 2009;119:1983–7.

12. Sawka AM, Brierley JD, Tsang RW, Thabane L, Rotstein L, Gafni A, et al. An updated systematic review and commentary examining the effectiveness of radioactive iodine remnant ablation in well-differentiated thyroid cancer. Endocrinol Metab Clin North Am 2008;37:457–80.
